# Prenatal and childhood predictors of hair cortisol concentration in mid-childhood and early adolescence

**DOI:** 10.1371/journal.pone.0228769

**Published:** 2020-02-04

**Authors:** Joshua Petimar, Sheryl L. Rifas-Shiman, Marie-France Hivert, Abby F. Fleisch, Henning Tiemeier, Emily Oken

**Affiliations:** 1 Department of Epidemiology, Harvard T.H. Chan School of Public Health, Boston, Massachusetts, United States of America; 2 Department of Population Medicine, Harvard Medical School and Harvard Pilgrim Health Care Institute, Boston, Massachusetts, United States of America; 3 Diabetes Unit, Massachusetts General Hospital, Boston, Massachusetts, United States of America; 4 Pediatric Endocrinology and Diabetes and Center for Outcomes Research and Evaluation, Maine Medical Center, Portland, Maine, United States of America; 5 Department of Social and Behavioral Sciences, Harvard T.H. Chan School of Public Health, Boston, Massachusetts, United States of America; 6 Erasmus University Medical Center-Sophia Children's Hospital, Rotterdam, Netherlands; 7 Department of Nutrition, Harvard T.H. Chan School of Public Health, Boston, Massachusetts, United States of America; University of Hong Kong, CHINA

## Abstract

**Background:**

Hair cortisol concentration (HCC) is an increasingly used measure of systemic cortisol concentration. However, determinants of HCC in children and adolescents are unclear because few prospective studies have been conducted to date.

**Study design:**

We followed 725 children in Project Viva, a pre-birth cohort study of mothers and children, who provided hair samples at mid-childhood (median age: 7.7 years) or early adolescence (median age: 12.9 years). We examined associations of various factors measured from pregnancy to mid-childhood with HCC in mid-childhood and early adolescence, as well as change in HCC between these time points (ΔHCC).

**Results:**

There were 426 children with HCC measurements in both mid-childhood and early adolescence, 173 children with measures only in mid-childhood, and 126 with measures only in early adolescence. HCC was lower in mid-childhood (median 1.0pg/mg [interquartile range, IQR: 0.5, 2.4]) than early adolescence (2.2pg/mg [1.1, 4.4]). In multivariable-adjusted regression models, female sex (β = -0.41, 95% CI: -0.67, -0.15) and birth weight-for-gestational age z-score (β = -0.19, 95% CI: -0.33, -0.04) were associated with lower mid-childhood HCC, while prenatal smoking was associated with higher mid-childhood HCC (β = 0.53, 95% CI: 0.04, 1.01). In early adolescence, child age (β = 0.34 per year, 95% CI: 0.21, 0.46) female sex (β = 0.33, 95% CI: 0.10, 0.57), and maternal pre-pregnancy body mass index (β = 0.15 per 5-kg/m^2^, 95% CI: 0.01, 0.29) were positively associated with HCC. Child anthropometric measures and biomarker concentrations were not associated with HCC.

**Conclusion:**

Maternal pre-pregnancy BMI, maternal prenatal smoking, and low birth weight were associated with higher mid-childhood and adolescent HCC. However, few postnatal characteristics were associated with HCC.

## Introduction

Hair cortisol concentration (HCC) is a relatively new measure of systemic cortisol concentration and hypothalamic-pituitary-adrenal (HPA) axis activity [1, 2]. In epidemiologic studies, HCC may be favored over other biomarkers of cortisol secretion (i.e. salivary, blood, or urinary cortisol) due to its relatively long time integration, ease of storage, robustness to acute stressors, and minimally invasive collection [2]. Moreover, in adults, the validity of HCC as a measure of HPA axis activity has been somewhat supported by studies observing higher HCC in individuals with Cushing’s disease [3, 4], chronic pain [5], cardiovascular disease [6], childhood trauma [7], schizophrenia and bipolar disorder [8], and post-traumatic stress disorder [9–11], though HCC has been less reliably associated with various sociodemographic stressors [2].

Although HCC is increasingly being used instead of other measures of cortisol secretion in epidemiologic investigations, few studies have examined HCC determinants in children and adolescents. Identifying HCC determinants in these populations is important to generate hypotheses about relationships between early life exposures and HPA axis activity as well as to adjust for confounders of associations of HCC with health outcomes. While some studies in children have observed positive associations of male sex and adiposity with HCC and inverse associations of household income and parental education with HCC [12], the current literature on HCC determinants is limited by a dearth of prospective studies, casting doubt on the directionality of some of these findings. Specifically, the bidirectional relationship between stress and many health outcomes [13] makes previous cross-sectional studies vulnerable to reverse causation. Furthermore, exposures during the prenatal and early postnatal periods may influence long-term chronic disease risk according to the developmental origins of health and disease hypothesis [14]. This hypothesis posits that prenatal and early life exposures may affect future health through inappropriate predictive adaptive responses (based on mismatched prenatal and postnatal environments) [15] or through direct exposure to environmental factors that increase risk of chronic disease [16]. These factors (e.g. birth weight and postnatal growth) have been understudied with relation to child and adolescent HCC [12] but may affect health later in life via cortisol and the HPA axis [17–19].

To address these gaps, we explored prospective associations of various sociodemographic, environmental, behavioral, and cardiometabolic exposures with HCC in mid-childhood and early adolescence in Project Viva, a prospective pre-birth cohort of mothers and children. We specifically examined characteristics that are predictive of obesity and poor cardiometabolic health in children and adolescents because we hypothesized that these factors would also be the most important determinants of HCC in this population.

## Materials and methods

### Study population

Project Viva is an ongoing prospective cohort that recruited women carrying a singleton pregnancy during their initial obstetric care visit at Atrius Harvard Vanguard Medical Associates between 1999 and 2002 in eastern Massachusetts [20]. Demographic, medical, lifestyle, and other health-related information on the cohort has been collected via annual in-person interviews and/or questionnaires since baseline [20]. Of 2128 live singleton births, 1260 attended an in-person research visit in mid-childhood (median age: 7.7 years; range: 6.6–10.7 years) or early adolescence (median age: 12.9 years; range: 11.9–16.6 years).

White children who provided hair samples at the mid-childhood (n = 639) or early adolescent in-person visits (n = 567) were eligible for the present study. We excluded non-white children because of differences in hair texture and hair growth rate by race/ethnicity [21], which may make HCC measurements across racial and ethnic groups incomparable. There were too few participants in strata of non-white racial/ethnic groups who provided hair samples to perform analyses stratified by race (n = 96 black participants; n = 40 Asian participants; n = 38 Hispanic participants). No mother reported that their child had been diagnosed with an HPA-related illness (e.g. Cushing’s disease, Addison’s disease). However, we did exclude children who had taken inhaled or oral steroids within one month of the mid-childhood or early adolescent visits (n = 25) because these medications may affect HCC measurements. After this exclusion, there were 599 children in the final analysis for mid-childhood HCC and 552 children in the final analysis for early adolescent HCC (n = 725 children in either analysis). There were 426 children with HCC measurements in both mid-childhood and early adolescence; there were 173 children with HCC measurements only in mid-childhood and 126 children with HCC measurements only in early adolescence. This study protocol was approved by the Institutional Review Board at Harvard Pilgrim Health Care. All mothers provided written informed consent at each visit.

### Hair collection and HCC assessment

We collected hair samples measuring 3cm in length from the posterior vertex region of the scalp of participants at the mid-childhood and early adolescent visits. Hair strands were cut as close as possible to the scalp, tied to identify the scalp end, and stored in a paper envelope away from light. For measurement of hair cortisol concentrations, lab personnel first washed the hair strands in isopropanol, and subsequently extracted cortisol using liquid chromatography tandem mass spectrometry [22]. We did not assess the intra-assay coefficient of variation (%CV) due to lack of duplicate hair samples, although the intra-assay %CV was less than 10% in previous studies using this method [22]. Based on an average hair growth rate of 1cm/month [23], 3cm of hair represents hair grown over approximately three months prior to collection, less the ~3 weeks of hair growth that has not yet emerged from the scalp. Only n = 19 mid-childhood HCC values (3%) and n = 11 early adolescent HCC values (2%) were undetectable; we kept these samples in our final analysis and assigned them a value of 0.01pg/mg, which was half of the lowest detectable HCC value.

### Parental and prenatal characteristics

Mothers reported their age, educational attainment, household income, pre-pregnancy weight, height, prenatal smoking (i.e. whether they smoked during pregnancy), and father’s weight and height via questionnaire and interview at recruitment. We calculated gestational weight gain by subtracting pre-pregnancy weight from the last recorded weight in clinical records in the four weeks prior to delivery. We then categorized this weight gain as excessive or not based on BMI category and Institute of Medicine guidelines [24].

### Child characteristics

We extracted data on infant birth date and sex from hospital medical records. We calculated gestational age by subtracting the date of the last menstrual period from the date of delivery. If gestational age according to second-trimester ultrasound differed from that according to the last menstrual period by more than ten days, we used the ultrasound result to determine gestational age. We collected infant birth weight from hospital medical records, and calculated birth weight-for-sex-and-gestational age z-scores using national reference data [25]. We used birth weight z-scores instead of raw birth weight values because birth weight is highly correlated with gestational age, and we wanted a measure of newborn size that was independent of time in utero. We collected information on infant sleep duration at the infancy in-person visit (median age: 6.3 months; range: 4.9–10.6 months) and on the one-year questionnaire, and averaged values from each time period to calculate mean infant sleep duration. We also collected information on breastfeeding duration on the one-year questionnaire. We measured infant weight using a digital scale (model 881; Seca, Hamburg, Germany), and length using a measuring board (Shorr Productions, Olney, MD) [26]. At in-person visits in early childhood (median age: 3.1 years; range: 2.9–6.0 years), mid-childhood, and early adolescence, we measured children’s weight using a calibrated scale (model TBF-300A; Tanita Corporation of America, Inc., Arlington Heights, IL) and height using a calibrated stadiometer (Shorr Productions, Olney, MD), from which we calculated BMI-for-age-and-sex z-scores [27], as well as waist circumference (cm) using a non-stretchable measuring tape (Hoechstmass Balzer GmbH, Sulzbach, Germany). We used z-scores over raw measurement values for BMI because in growing children, BMI varies by age and sex. Comparison of BMI to a reference population of the same age and sex is thus necessary to determine whether a child has underweight, healthy weight, overweight, or obesity [28].

At the mid-childhood visit, we collected an 8-hour fasting blood sample. All samples were immediately refrigerated, processed within 24 hours, and stored at –80°C until time of analysis. These samples were used to measure glucose and insulin (from which HOMA-IR was calculated), adiponectin, high-density lipoprotein (HDL), triglycerides, C-reactive protein (CRP), interleukin-6 (IL-6), and leptin. Systolic blood pressure (SBP) measurements were taken five times using biannually-calibrated automated oscillometric monitors (Dinamap Pro100, Tampa, Florida), from which we calculated mean SBP. HOMA-IR, HDL, SBP, triglycerides, and waist circumference were used to create a metabolic risk z-score, as described previously [29].

At the mid-childhood visit, mothers reported children’s dietary behaviors on a PrimeScreen that assessed intake of 18 food groups (whole grains, vegetables, fruits, dairy, meat, snacks, and beverages), which we used to calculate Youth Healthy Eating Index scores as described previously [30]. Mothers also reported how many hours their child engaged in weekly vigorous physical activity, children’s secondhand smoke exposure (at home or outside of the home), and oral or inhaled steroid use over the past month via questionnaire and interviews. Children self-reported pubertal development via the Pubertal Development Scale [31], which has been moderately correlated with physician Tanner staging [32]. Lastly, mothers reported whether their children were ever diagnosed with any medical conditions in interviews at the mid-childhood visit; we considered children with attention deficit/hyperactive disorder (n = 6), congenital heart disease (n = 4), autism (n = 3), chromosomal disorders (n = 2), inflammatory bowel disease (n = 1), diabetes (n = 1), cancer (n = 1), and juvenile rheumatoid arthritis (n = 1) to have a chronic illness. We considered this a potential predictor of early adolescent HCC because, despite having different biology, these conditions could all be associated with stress.

### Statistical analysis

We performed all analyses using SAS version 9.4 (Cary, NC). We used multivariable linear regression to estimate associations of various sociodemographic, environmental, behavioral, and anthropometric characteristics of children and their parents with mid-childhood and early adolescent HCC. We selected characteristics that we hypothesized would be associated with HCC (i.e. variables likely to be associated with stress and/or adiposity). Our primary analyses examined associations of these variables with mid-childhood HCC and early adolescent HCC separately. In secondary analyses, we explored associations between these variables with change in HCC from mid-childhood to early adolescence (ΔHCC). We log-transformed HCC values in all analyses, which greatly improved normality upon visual inspection. We also log-transformed HOMA-IR, CRP, IL-6, and leptin values, which improved normality.

For all analyses, we first built models that included only demographic characteristics of children and their parents as covariates (Model 1). We next built models that examined prenatal (Model 2), infant (Model 3), and early-childhood (Model 4) characteristics. Models 1–4 were examined in relationship to mid-childhood HCC and early adolescent HCC separately. We then examined associations of mid-childhood lifestyle characteristics, anthropometric measures, and biomarkers (Models 5, 6, and 7, respectively) with early adolescent HCC only. Each model was adjusted for hypothesized confounders of the variables and HCC measure of interest. We did not include multiple anthropometric or biomarker measures in the same model because these measures are often highly correlated and including them in the same model can change the interpretation of the main variable of interest.

We also examined cross-sectional associations of mid-childhood characteristics with mid-childhood HCC, as well as of early adolescent anthropometric measures with early adolescent HCC, but these were considered exploratory because the exposures and outcomes occurred at the same time. We used multiple imputation to impute values of missing data for all exposures and covariates in all analyses. We did this by imputing 50 values for each missing observation and then combining the multivariable modeling estimates using PROC MIANALYZE. We calculated the range of R^2^ values across imputed datasets for each model in our primary analyses. We reinterpreted our results after controlling for the false discovery rate (FDR) at q = 0.10 using the linear step up method of Benjamini and Hochberg [33].

For all analyses, we examined associations in males and females combined as well as in strata of sex, since we expected more females than males to have experienced onset of puberty at the time of early adolescent hair collection, which could influence cortisol secretion [34]. We tested for interaction by sex by evaluating the P-value of an interaction term between the exposure of interest and sex in the combined models. We calculated 2-sided 95% confidence intervals (CI) for all statistical tests.

## Results

Compared to White children who participated in Project Viva but did not provide a hair sample at either visit, those who provided at least one hair sample (and were thus eligible for the current study), were more likely to be female, have a mother who graduated from college, and have been breastfed for one year or longer. Included participants were also less likely to have a mother who smoked during pregnancy or to be exposed to second-hand smoke in mid-childhood (**Table 1**). The median HCC was 0.98 (interquartile range, IQR: 0.49, 2.44) (log-transformed: -0.03 [-0.71, 0.88]) in mid-childhood and 2.21 (1.13, 4.44) (log-transformed: 0.78 [0.12, 1.48]) in early adolescence. The median ΔHCC was 0.78 (-0.51, 2.69).

**Table 1:** Characteristics^a^ of White Project Viva participants who provided and did not provide at least one hair sample.

		Did not provide hair sample	Provided at least one hair sample
Characteristic		(n = 592)	(n = 725)
Mid-childhood HCC^b^ (pg/mg)		--	0.98 (0.49, 2.44)
Early adolescent HCC^b^ (pg/mg)		--	2.21 (1.13, 4.44)
Age at mid-child hair collection (years)		--	7.9 (0.8)
Age at early teen hair collection (years)		--	13.2 (0.9)
Female (%)		45	54
**Parental characteristics**			
	Maternal age (years)	32.2 (4.5)	33.5 (4.1)
	Maternal pre-pregnancy BMI (kg/m^2^)	24.3 (5.2)	24.1 (4.5)
	Excessive pregnancy weight gain^c^ (%)	61	61
	Paternal BMI (kg/m^2^)	26.5 (3.8)	26.4 (3.8)
	Yearly household income (> vs. ≤$70,000) (%)	67	73
	Mother's education (college graduate vs. not a college graduate) (%)	67	82
	Mother smoked during pregnancy (%)	17	8
**Early life characteristics**			
	Birthweight-for-sex-and-gestational age z-score	0.25 (0.98)	0.32 (0.92)
	Gestational age (weeks)	39.6 (1.8)	39.7 (1.6)
	Breastfed ≥12 months (%)	17	31
	Infant sleep duration (hours/day)	12.6 (1.7)	12.7 (1.4)
**Early-childhood characteristics**			
	BMI-for-age-and-sex z-score	0.45 (1.31)	0.44 (1.01)
	Waist circumference (cm)	51.3 (4.8)	51.3 (3.6)
	Height (cm)	97.6 (6.1)	96.9 (4.4)
	Waist-height ratio	0.53 (0.05)	0.53 (0.03)
**Mid-childhood characteristics**			
	Vigorous physical activity (hours/week)	3.8 (5.8)	3.6 (3.8)
	Youth Healthy Eating Index score	58.5 (15.7)	60.0 (10.9)
	Secondhand smoke exposure (%)	15	10
	Puberty development score	1.1 (0.4)	1.1 (0.3)
	Chronic illness^d^	1	3
**Mid-childhood anthropometry**			
	BMI-for-age-and-sex z-score	0.34 (1.32)	0.30 (0.96)
	Waist circumference (cm)	59.3 (10.5)	59.4 (7.6)
	Height (cm)	128.8 (13.2)	128.3 (7.8)
	Waist-height ratio	0.46 (0.07)	0.46 (0.05)
**Mid-childhood biomarkers**			
	Metabolic risk z-score	0.05 (0.92)	0.01 (0.72)
	Systolic blood pressure (mm Hg)	95.1 (14.1)	94.7 (9.1)
	Adiponectin (μg/ml)	15.9 (14.6)	15.8 (11.9)
	HOMA-IR	1.4 (1.7)	1.5 (1.3)
	HDL (mg/dL)	55.0 (19.9)	55.6 (17.1)
	CRP (mg/L)	0.6 (2.7)	0.6 (2.0)
	IL-6 (pg/mL)	1.0 (2.0)	1.0 (1.7)
	Leptin (ng/mL)	5.2 (9.6)	5.3 (7.6)
	Triglycerides (mg/dL)	66.3 (59.2)	64.0 (46.6)

^a^Mean (SD) or % presented unless otherwise stated

^b^Median and interquartile range presented

^c^As defined by Institute of Medicine Weight Gain Recommendations for Pregnancy

^d^Includes attention deficit/hyperactive disorder (n = 6), heart disease (n = 4), autism (n = 3), chromosomal disorders (n = 2), inflammatory bowel disease (n = 1), diabetes (n = 1), cancer (n = 1), and juvenile rheumatoid arthritis (n = 1)

In mid-childhood (**Table 2**), females had lower log-transformed HCC than males (β = –0.41, 95% CI: –0.67, –0.15). We also found that maternal prenatal smoking (β = 0.53 for prenatal smoking vs. no prenatal smoking, 95% CI: 0.04, 1.01), and lower birth weight-for-sex-and-gestational age z-score (β = –0.19 per 1-unit increment, 95% CI: –0.33, –0.04) were associated with higher HCC. Higher BMI z-score, waist circumference, and height in early childhood were associated with higher HCC in mid-childhood, but all CIs included the null. When controlling for the FDR at q = 0.10, we rejected null hypotheses for associations of sex and birth weight-for-sex-and-gestational age z-score. R^2^ values in the imputed datasets for the most complex model (Model 4) ranged from 0.05 to 0.06. We did not observe heterogeneity by sex for any associations with mid-childhood HCC (**S1 Table**). In cross-sectional analyses in mid-childhood, we did not observe associations of any measures of anthropometry, physical activity, diet quality, or secondhand smoke exposure with HCC, except for minor elevations in HCC associated with BMI z-score and waist-height ratio, though all CIs included the null (**S2 Table)**.

**Table 2:** Associations (β [95% CI]^i^) of prenatal, parental, and child characteristics with hair cortisol concentration^a^ in White children.

		Mid-childhood HCC (n = 599)	Early adolescent HCC (n = 552)
**Model 1: demographic characteristics**			
	Age (per year)	0.09 (-0.08, 0.26)	0.34 (0.21, 0.46)
	Female	-0.41 (-0.67, -0.15)	0.33 (0.10, 0.57)
	Yearly household income (≥ vs. <$70,000)	-0.04 (-0.34, 0.27)	-0.11 (-0.37, 0.16)^i^
	Mother's education (college graduate vs. not a college graduate)	-0.15 (-0.49, 0.19)	0.15 (-0.17, 0.47)^j^
**Model 2: prenatal characteristics**^**b**^			
	Maternal age (per 5 years)	0.12 (-0.04, 0.27)	-0.09 (-0.24, 0.06)
	Maternal pre-pregnancy BMI (per 5kg/m^2^)	0.11 (-0.04, 0.27)	0.15 (0.01, 0.29)
	Excessive pregnancy weight gain	-0.10 (-0.37, 0.17)	0.01 (-0.23, 0.26)
	Mother smoked during pregnancy	0.53 (0.04, 1.01)	-0.17 (-0.61, 0.27)
	Paternal BMI (per 5kg/m^2^)	0.06 (-0.12, 0.24)	-0.08 (-0.24, 0.08)
**Model 3: early life characteristics**^**c**^			
	Gestational age (per week)	-0.01 (-0.09, 0.07)	0.06 (-0.01, 0.13)
	Birthweight-for-sex-and-gestational age z-score	-0.19 (-0.33, -0.04)	-0.05 (-0.18, 0.09)
	Breastfed ≥12 months	-0.08 (-0.38, 0.21)	-0.03 (-0.30, 0.24)
	Infant sleep duration (per hour/day)	0.05 (-0.05, 0.15)	-0.03 (-0.13, 0.06)
**Model 4: early-childhood anthropometry**^**d**^			
	BMI-for-age-and-sex z-score	0.05 (-0.09, 0.19)	0.09 (-0.04, 0.21)
	Waist circumference (per 5cm)	0.03 (-0.17, 0.22)	0.09 (-0.10, 0.27)
	Height (per 5cm)	0.10 (-0.07, 0.28)	0.06 (-0.09, 0.21)
	Waist-height ratio (per 0.1 units)	-0.12 (-0.54, 0.31)	0.08 (-0.29, 0.45)
**Model 5: mid-childhood characteristics**^**e**^			
	Vigorous physical activity (per 5 hours/week)	--	0.07 (-0.10, 0.23)
	Youth Healthy Eating Index score (per 10 points)	--	-0.08 (-0.19, 0.04)
	Secondhand smoke exposure (%)	--	0.15 (-0.25, 0.56)
	Puberty development score	--	0.12 (-0.36, 0.60)
	Chronic illness^f^	--	0.61 (-0.05, 1.27)
**Model 6: mid-childhood anthropometry**^**g**^			
	BMI-for-age-and-sex z-score	--	0.11 (-0.03, 0.25)
	Waist circumference (per 5cm)	--	0.06 (-0.04, 0.16)
	Height (per 5cm)	--	0.05 (-0.04, 0.15)
	Waist-height ratio (per 0.1 units)	--	0.10 (-0.20, 0.40)
**Model 7: mid-childhood biomarkers**^**h**^			
	Metabolic risk z-score	--	0.00 (-0.32, 0.32)
	Systolic blood pressure (per 10mm Hg)	--	-0.08 (-0.24, 0.09)
	Adiponectin (μg/ml)	--	0.01 (-0.01, 0.02)
	HOMA-IR^a^	--	0.06 (-0.17, 0.29)
	HDL (mg/dL)	--	0.00 (-0.01, 0.01)
	CRP (mg/L)^a^	--	-0.05 (-0.14, 0.04)
	IL-6 (pg/mL)^a^	--	-0.02 (-0.18, 0.14)
	Leptin (ng/mL)^a^	--	-0.04 (-0.24, 0.16)
	Triglycerides (per 10 mg/dL)	--	0.03 (-0.03, 0.08)

^a^Natural log-transformed

^b^Model includes all variables in model 1 as well as all prenatal characteristics

^c^Model includes all variables in models 1 and 2, as well as all early life characteristics

^d^Model includes all variables in models 1, 2, and 3. Each anthropometric measure was included in a separate model

^e^Model includes all variables in models 1, 2, and 3, as well as all mid-childhood characteristics. Mid-childhood BMI-for-age-and-sex z-score was also included in the model.

^f^Includes attention deficit/hyperactive disorder (n = 6), heart disease (n = 4), autism (n = 3), chromosomal disorders (n = 2), inflammatory bowel disease (n = 1), diabetes (n = 1), cancer (n = 1), and juvenile rheumatoid arthritis (n = 1)

^g^Model includes all variables in models 1, 2, and 3, and 5. Each anthropometric measure was included in a separate model.

^h^Model includes all variables in models 1, 2, 3, and 5, as well as mid-childhood BMI-for-age-and-sex z-score. Each biomarker was included in a separate model.

^i^Significant difference (P-interaction = 0.01) between males (β = 0.24, 95% CI: -0.20, 0.69) and females (β = -0.35, 95% CI: -0.67, -0.02)

^j^Significant difference (P-interaction = 0.03) between males (β = 0.43, 95% CI: -0.06, 0.93) and females (β = -0.12, 95% CI: -0.53, 0.29)

In early adolescence (**Table 2**), log HCC was higher in older children (β = 0.34 per 1-year increment in age, 95% CI: 0.21, 0.46) and in females (β = 0.33, 95% CI: 0.10, 0.57), opposite to the association we observed in mid-childhood. Higher maternal pre-pregnancy BMI was associated with higher adolescent HCC (β = 0.15 per 5-kg/m^2^ increment, 95% CI: 0.01, 0.29). We did not observe associations of any other early-childhood or mid-childhood factors with early adolescent HCC, though having a chronic illness was associated with marginally higher HCC (β = 0.61, 95% CI: -0.05, 1.27). When controlling for the FDR at q = 0.10, we rejected null hypotheses for associations of age and sex. R^2^ values in the imputed datasets for the most complex model (Model 5) ranged from 0.09 to 0.11. We observed heterogeneity by sex for associations of annual household income (in males: β = 0.24, 95% CI: –0.20, 0.69; in females: β = –0.35 for >$70,000 vs. ≤$70,000, 95% CI: –0.67, –0.02; P-interaction = 0.01), and maternal education (in females: β = –0.12, 95% CI: –0.53, 0.29; in males: β = 0.43, 95% CI: –0.06, 0.93; P-heterogeneity = 0.03) with early adolescent HCC (**S1 Table**). We also did not observe associations between early adolescent anthropometric measures and early adolescent HCC (**S3 Table**).

We observed that ΔHCC from mid-childhood to early adolescence was greater in children who were older in mid-childhood (β = 0.23 per 1-year increment in age, 95% CI: 0.01, 0.45), in females (β = 0.48, 95% CI: 0.14, 0.82), and in those with a chronic illness (β = 1.05, 95% CI: 0.03, 2.07) (**Table 3**). However, ΔHCC was lower in children whose mothers smoked during pregnancy (β = –0.82, 95% CI: –1.45, –0.20). Other parent and child characteristics were not associated with ΔHCC from mid-childhood to early adolescence. We observed heterogeneity between males and females for the association of household income with ΔHCC (in males: β = 0.20, 95% CI: -0.47, 0.87; in females: β = –0.69, 95% CI: –1.17, –0.22; P-heterogeneity = 0.03) **(S4 Table**).

**Table 3:** Associations (β [95% CI]) of prenatal, parental, and child characteristics with change in hair cortisol concentration^a^ from mid-childhood to early adolescence in White children (n = 426).

**Model 1: demographic characteristics**		
	Child age (per year)	0.23 (0.01, 0.45)
	Female	0.48 (0.14, 0.82)
	Yearly household income (≥ vs. <$70,000)	-0.35 (-0.74, 0.04)^i^
	Mother's education (college graduate vs. not a college graduate)	-0.05 (-0.51, 0.40)
**Model 2: prenatal characteristics**^**b**^		
	Maternal age (per 5 years)	0.08 (-0.13, 0.30)
	Maternal pre-pregnancy BMI (per 5kg/m^2^)	0.03 (-0.16, 0.23)
	Excessive pregnancy weight gain	0.07 (-0.28, 0.43)
	Mother smoked during pregnancy	-0.82 (-1.45, -0.20)
	Paternal BMI (per 5kg/m^2^)	-0.03 (-0.26, 0.20)
**Model 3: early life characteristics**^**c**^		
	Gestational age (per week)	0.02 (-0.09, 0.14)
	Birthweight-for-sex-and-gestational age z-score	0.02 (-0.17, 0.21)
	Breastfed ≥12 months	0.07 (-0.31, 0.46)
	Infant sleep duration (per hour/day)	0.01 (-0.13, 0.14)
**Model 4: early-childhood anthropometry**^**d**^		
	BMI-for-age-and-sex z-score	0.01 (-0.17, 0.19)
	Waist circumference (per 5cm)	0.02 (-0.24, 0.28)
	Height (per 5cm)	0.14 (-0.08, 0.37)
	Waist-height ratio (per 0.1 units)	-0.16 (-0.71, 0.38)
**Model 5: mid-childhood characteristics**^**e**^		
	Vigorous physical activity (per 5 hours/week)	-0.15 (-0.40, 0.10)
	Youth Healthy Eating Index score (per 10 points)	-0.05 (-0.23, 0.13)
	Secondhand smoke exposure (%)	0.51 (-0.11, 1.14)
	Puberty development score	0.09 (-0.59, 0.76)
	Chronic illness^f^	1.05 (0.03, 2.07)
**Model 6: mid-childhood anthropometry**^**g**^		
	BMI-for-age-and-sex z-score	-0.01 (-0.21, 0.20)
	Waist circumference (per 5cm)	-0.08 (-0.23, 0.08)
	Height (per 5cm)	0.11 (-0.05, 0.27)
	Waist-height ratio (per 0.1 units)	-0.36 (-0.79, 0.07)
**Model 7: mid-childhood biomarkers**^**h**^		
	Metabolic risk z-score	0.02 (-0.40, 0.44)
	Systolic blood pressure (per 10mm Hg)	0.02 (-0.21, 0.25)
	Adiponectin (μg/ml)	-0.01 (-0.03, 0.02)
	HOMA-IR^a^	0.01 (-0.32, 0.34)
	HDL (mg/dL)	0.00 (-0.01, 0.02)
	CRP (mg/L)^a^	-0.06 (-0.19, 0.07)
	IL-6 (pg/mL)^a^	-0.08 (-0.32, 0.17)
	Leptin (ng/mL)^a^	-0.07 (-0.35, 0.21)
	Triglycerides (per 10 mg/dL)	0.04 (-0.04, 0.11)

^a^Natural log-transformed

^b^Model includes all variables in model 1 as well as all prenatal characteristics

^c^Model includes all variables in models 1 and 2, as well as all early life characteristics

^d^Model includes all variables in models 1, 2, and 3. Each early-childhood anthropometric measure was included in a separate model

^e^Model includes all variables in models 1, 2, and 3, as well as all mid-childhood characteristics. Mid-childhood BMI-for-age-and-sex z-score was also included in the model.

^f^Includes attention deficit/hyperactive disorder (n = 3), heart disease (n = 3), chromosomal disorders (n = 2), autism (n = 1), inflammatory bowel disease (n = 1), diabetes (n = 1), cancer (n = 1), and juvenile rheumatoid arthritis (n = 1)

^g^Model includes all variables in models 1, 2, and 3, and 5. Each anthropometric measure was included in a separate model.

^h^Model includes all variables in models 1, 2, 3, and 5, as well as mid-childhood BMI-for-age-and-sex z-score. Each biomarker was included in a separate model.

^i^Significant difference (P-interaction = 0.03) between males (β = 0.20, 95% CI: -0.47, 0.87) and females (β = -0.69, 95% CI: -1.17, -0.22)

## Discussion

In this analysis of a prospective pre-birth cohort of mothers and children, we found that child age, sex, and some prenatal factors, including maternal pre-pregnancy BMI, maternal prenatal smoking, and low birth weight, were associated with higher mid-childhood or early adolescent HCC. However, few postnatal environmental, lifestyle, or cardiometabolic characteristics were associated with HCC at either time point, or ΔHCC between the two time points.

Maternal prenatal smoking [35] and pre-pregnancy adiposity [36] have each been identified as likely regulators of fetal programming, are known to adversely affect the fetal environment, and have been proposed to influence HPA axis function [37, 38]. Our study, which observed associations between prenatal smoking and mid-childhood HCC, as well as between pre-pregnancy BMI and early adolescent HCC, is therefore consistent with the hypothesis that conditions of the fetal environment are important for future HPA axis activity. This is additionally supported by the fact that birth weight, a commonly used proxy of the fetal environment [39], was associated with mid-childhood HCC even after adjusting for prenatal smoking and pre-pregnancy BMI. Other studies have found associations of prenatal smoking and birth weight with cortisol secretion in infants and young children [40, 41], lending support to our findings. However, in our study, associations of prenatal smoking and low birth weight with HCC did not persist in early adolescence, raising questions about whether these associations attenuate over time. Conversely, pre-pregnancy BMI was not associated with mid-childhood HCC. Explanation for the inconsistency of these associations over time is unclear; however, because this study is the first to our knowledge to examine these factors with HCC at different time points, additional studies investigating these associations are warranted. Moreover, future studies investigating HCC in relation to health outcomes should adjust for these early life characteristics because they may confound these associations.

While there is great interest in HCC as a biomarker of stress, previous studies have yielded conflicting results for associations between traditional socioeconomic indicators of stress (e.g. low household income and parental education) with HCC. Indeed, some studies have observed the expected inverse associations between parental education and HCC in young children [42, 43], whereas other studies observed no association [41, 44] or a weakly positive association [45]. Results for household income have been similarly mixed [43, 46]. While we observed lower prenatal household income to be associated with higher early adolescent HCC and ΔHCC in females, we did not observe associations between these indicators with mid-childhood HCC in females or with HCC in males in either period. Whether socioeconomic stress is indeed inversely associated with HCC (and whether any such association differs by sex) thus remains unclear from the present study. Importantly, approximately 75% of the children in our study lived in households with a yearly income ≥$70,000, over 80% had a mother who graduated from college, and all had health insurance at the time of recruitment. Because our study participants generally came from high socioeconomic environments, we were unable to examine strong contrasts in household income or maternal education, which may have reduced our ability to observe associations between these indicators and HCC. The associations we observed of having a chronic illness in mid-childhood with early adolescent HCC and with ΔHCC may suggest a potential role of mental or biological stress on HCC. However, because this definition included many heterogenous diseases, which could have different effects on stress and on HPA-related pathways, associations of each type of chronic condition with HCC should be explored in greater detail in future studies. Given the small number of children in our study whose mothers reported any one particular disease, we could not explore this in the current study.

The models we built explained a small amount of the variability in HCC, though the variables explained more variability in early adolescent HCC than mid-childhood HCC. The limited ability of these characteristics to account for the variability of HCC in children has been observed previously [12] and may explain the few associations we observed of child characteristics with HCC. We did, however, observe associations of sex and age with HCC. Girls had lower mid-childhood HCC but higher early adolescent HCC than boys. While some previous studies have observed higher HCC in pre-pubertal boys [44, 46], a recent review found most studies reported no difference in HCC by sex among adolescents [12]. However, at least one study has observed higher salivary cortisol levels in post-pubertal girls than post-pubertal boys [47], and salivary cortisol has been observed to be higher in adult women than men [48]. The differences we observed between males and females over time may be due to pubertal onset, which occurs earlier in females than males and is associated with increased cortisol concentration [34]. This may also explain the positive association we observed for age and HCC in early adolescence; conversely, in mid-childhood, very few participants had experienced signs of pubertal onset, which may explain the lack of association we observed at this time point. We did not observe associations between any anthropometric measures and any HCC outcomes. This is in contrast to some previous studies that observed positive associations of BMI or waist circumference with HCC in childhood [44–46], although our analyses were prospective and adjusted for more potential confounding variables than previous studies, which may explain some of these discrepancies. Moreover, other studies also did not observe any associations between these anthropometric measures and HCC in children [49, 50]. We also did not observe any prospective or cross-sectional associations of overall metabolic risk with HCC outcomes, which is generally consistent with previous studies that examined some of these relationships using hair, urinary, or salivary cortisol [44, 51, 52]. Our results therefore suggest that adiposity and cardiometabolic health may not be strong determinants of HCC in children.

We conducted many analyses in this study, implying that some of the associations we observed may be due to chance. This is supported by the fact that prenatal smoking and maternal pre-pregnancy BMI were not associated with HCC after controlling for the FDR at q = 0.10. However, our primary aim was to generate hypotheses regarding possible determinants of HCC in children and to identify potential confounders in future analyses of HCC and health outcomes. We therefore still consider prenatal smoking and maternal pre-pregnancy BMI possible determinants of HCC and believe that they should be considered as potential confounders in analyses that involve HCC. Future studies confirming and investigating the precise biological mechanisms underlying these associations are warranted.

Strengths of this study include its prospective nature, long follow-up, collection of hair samples over two time points, which allowed us to examine associations in both mid-childhood and early adolescence (as well change in HCC between these time periods), and detailed collection of prenatal, environmental, lifestyle, and anthropometric information, which allowed us to examine relationships with HCC that have not been previously reported, as well as adjust for many likely confounders of these associations. However, our study has limitations as well. First, because of differences in hair growth and texture between racial and ethnic groups [21], we analyzed all associations within strata of race/ethnicity. However, few black, Hispanic, and Asian participants provided hair samples, and so we excluded them from our analyses. Because stress experiences may differ between racial/ethnic groups in the US [53], our results may be generalizable only to white children. Second, most participants in this study lived in households with relatively high socioeconomic status, which further reduces the generalizability of our findings. Third, we included many variables in our models that could be related to one another, possibly making point estimates more sensitive to modeling assumptions and reducing our statistical power to find associations. However, variables that were most highly intercorrelated (i.e. adiposity measures taken at the same visit and biomarkers) were not included in the same model, likely avoiding substantial decreases in statistical power due to collinearity. Fourth, we did not have duplicate hair samples, and so could not assess the intra-assay %CV in our sample (though it has been reported as <10% in other populations) [22]. Fifth, we excluded children who had taken steroids in the month before hair collection, but the 3cm of hair we collected represented HCC over a period of three months. If some children took steroids 1–3 months before the hair collection, their HCC values could have been affected by this exposure. Sixth, we did not have information on participants’ medication use or trauma, which could be important determinants of HCC. Lastly, we did not have a measure of HCC prior to the mid-childhood visit, which may have led to unmeasured confounding of some associations. Early life HCC is likely associated with HCC later in life and could also be associated with other factors in mid-childhood or early adolescence (e.g. lifestyle characteristics, adiposity, etc.), thereby potentially confounding those (null) associations. However, most of the non-null associations we observed involved factors from the prenatal period, which cannot be confounded by early life HCC.

In summary, this study supports associations of maternal prenatal smoking, pre-pregnancy BMI, and low birth weight with higher childhood and adolescent HCC, but we overall observed few other associations for childhood socioeconomic, environmental, lifestyle, and anthropometric characteristics. Further studies are necessary to confirm the relationship of prenatal, parental, and childhood factors with HCC. Nevertheless, age, sex, birth weight-for-gestational-age z-score, maternal prenatal smoking, pre-pregnancy BMI, household income, and parental education may be determinants of HCC, and should be accounted for in analyses of HCC with childhood and adolescent outcomes.

## Supporting information

S1 Table Associations (β [95% CI]) of prenatal, parental, and child characteristics with hair cortisol concentration in White children by sex. (DOCX)

S2 Table Cross-sectional predictors of mid-childhood hair cortisol concentration in White children. (DOCX)

S3 Table Cross-sectional predictors of early adolescent hair cortisol concentration in White children (n = 552). (DOCX)

S4 Table Associations (β [95% CI]) of characteristics with change in hair cortisol concentration from mid-childhood to early adolescence in White children (n = 426) by sex. (DOCX)
