# Supplementary material for: Prenatal and childhood predictors of hair cortisol concentration in mid-childhood and early adolescence
Source: PLoS One. 2020 Feb 4;15(2):e0228769. doi: 10.1371/journal.pone.0228769 (PMC6999889; doi:10.1371/journal.pone.0228769)
Supplement: S1 Table — (DOCX) [file pone.0228769.s001.docx]

| **S1 Table. Associations (β [95% CI]) of prenatal, parental, and child characteristics with hair cortisol concentration^a^ in White children by sex** | | | | | | | | |
| --- | --- | --- | --- | --- | --- | --- | --- | --- |
|  |  | Mid-childhood HCC (n=599) | | |  | Early adolescent HCC (n=552) | | |
| Characteristic | | Males (n=273) | Females (n=326) | P-interaction^b^ |  | Males (n=241) | Females (n=311) | P-interaction^b^ |
| **Model 1: Demographic Characteristics** | |  |  |  |  |  |  |  |
|  | Age (per year) | 0.15 (-0.10, 0.39) | 0.05 (-0.18, 0.29) | 0.74 |  | 0.28 (0.09, 0.47) | 0.37 (0.20, 0.54) | 0.38 |
|  | Female | -- | -- |  |  | -- | -- |  |
|  | Yearly household income (≥ vs. <$70,000) | 0.01 (-0.45, 0.44) | -0.04 (-0.45, 0.38) | 0.66 |  | 0.24 (-0.20, 0.69) | -0.35 (-0.67, -0.02) | 0.01 |
|  | Mother's education (college graduate vs. not a college graduate) | 0.16 (-0.31, 0.63) | -0.44 (-0.93, 0.04) | 0.08 |  | 0.43 (-0.06, 0.93) | -0.12 (-0.53, 0.29) | 0.03 |
| **Model 2: Prenatal Characteristics^c^** | |  |  |  |  |  |  |  |
|  | Maternal age (per 5 years) | 0.18 (-0.04, 0.39) | 0.06 (-0.16, 0.28) | 0.31 |  | -0.14 (-0.38, 0.10) | -0.05 (-0.24, 0.14) | 0.75 |
|  | Maternal pre-pregnancy BMI (per 5kg/m^2^) | 0.07 (-0.17, 0.31) | 0.15 (-0.05, 0.35) | 0.54 |  | 0.15 (-0.11, 0.42) | 0.16 (0.01, 0.32) | 0.58 |
|  | Excessive pregnancy weight gain | -0.10 (-0.52, 0.31) | -0.09 (-0.46, 0.28) | 0.75 |  | 0.18 (-0.24, 0.60) | -0.08 (-0.38, 0.22) | 0.27 |
|  | Mother smoked during pregnancy | 0.43 (-0.22, 1.09) | 0.70 (-0.04, 1.44) | 0.38 |  | 0.03 (-0.61, 0.66) | -0.35 (-0.97, 0.27) | 0.58 |
|  | Paternal BMI (per 5kg/m^2^) | 0.12 (-0.13, 0.37) | 0.01 (-0.26, 0.27) | 0.79 |  | -0.11 (-0.36, 0.14) | -0.08 (-0.29, 0.14) | 0.61 |
| **Model 3: Early Life Characteristics^d^** | |  |  |  |  |  |  |  |
|  | Gestational age (per week) | 0.00 (-0.12, 0.11) | -0.03 (-0.15, 0.10) | 0.93 |  | 0.03 (-0.09, 0.15) | 0.08 (-0.02, 0.17) | 0.48 |
|  | Birthweight-for-sex-and-gestational age z-score | -0.32 (-0.52, -0.11) | -0.08 (-0.29, 0.13) | 0.11 |  | -0.01 (-0.23, 0.21) | -0.06 (-0.22, 0.11) | 0.82 |
|  | Breastfed ≥12 months | -0.17 (-0.59, 0.25) | 0.00 (-0.42, 0.43) | 0.86 |  | -0.29 (-0.73, 0.15) | 0.23 (-0.11, 0.57) | 0.04 |
|  | Infant sleep duration (per hour/day) | 0.00 (-0.14, 0.14) | 0.08 (-0.07, 0.22) | 0.48 |  | 0.03 (-0.13, 0.20) | -0.06 (-0.18, 0.05) | 0.15 |
| **Model 4: Early-Childhood Characteristics^e^** | |  |  |  |  |  |  |  |
|  | BMI-for-age-and-sex z-score | 0.09 (-0.11, 0.29) | 0.05 (-0.15, 0.25) | 0.92 |  | 0.06 (-0.15, 0.27) | 0.09 (-0.07, 0.25) | 0.93 |
|  | Waist circumference (per 5cm) | 0.13 (-0.15, 0.40) | -0.04 (-0.34, 0.25) | 0.77 |  | 0.10 (-0.19, 0.39) | 0.05 (-0.18, 0.29) | 0.71 |
|  | Height (per 5cm) | 0.06 (-0.19, 0.31) | 0.17 (-0.08, 0.42) | 0.28 |  | 0.03 (-0.21, 0.26) | 0.16 (-0.05, 0.36) | 0.39 |
|  | Waist-height ratio (per 0.1 units) | 0.18 (-0.41, 0.77) | -0.36 (-0.98, 0.26) | 0.30 |  | 0.16 (-0.43, 0.75) | -0.13 (-0.62, 0.37) | 0.35 |
| **Model 5: Mid-Childhood Characteristics^f^** | |  |  |  |  |  |  |  |
|  | Vigorous physical activity (per 5 hours/week) | -- | -- | -- |  | 0.30 (0.01, 0.59) | -0.07 (-0.27, 0.13) | 0.02 |
|  | Youth Healthy Eating Index score (per 10 points) | -- | -- | -- |  | 0.03 (-0.17, 0.24) | -0.11 (-0.26, 0.03) | 0.38 |
|  | Secondhand smoke exposure (%) | -- | -- | -- |  | 0.32 (-0.26, 0.89) | -0.14 (-0.72, 0.45) | 0.27 |
|  | Puberty development score | -- | -- | -- |  | -0.47 (-1.85, 0.92) | 0.26 (-0.23, 0.75) | 0.41 |
|  | Chronic illness^g^ | -- | -- | -- |  | 0.57 (-0.30, 1.44) | 0.69 (-0.47, 1.84) | 0.59 |
| **Model 6: Mid-Childhood Anthropometry^h^** | |  |  |  |  |  |  |  |
|  | BMI-for-age-and-sex z-score | -- | -- | -- |  | 0.10 (-0.14, 0.34) | 0.12 (-0.06, 0.30) | 0.99 |
|  | Waist circumference (per 5cm) | -- | -- | -- |  | 0.07 (-0.09, 0.23) | 0.06 (-0.08, 0.19) | 0.93 |
|  | Height (per 5cm) | -- | -- | -- |  | 0.04 (-0.11, 0.19) | 0.09 (-0.03, 0.21) | 0.51 |
|  | Waist-height ratio (per 0.1 units) | -- | -- | -- |  | 0.17 (-0.32, 0.67) | 0.02 (-0.36, 0.40) | 0.73 |
| **Model 7: Mid-Childhood Biomarkers^i^** | |  |  |  |  |  |  |  |
|  | Metabolic risk z-score | -- | -- | -- |  | -0.18 (-0.72, 0.35) | 0.12 (-0.24, 0.47) | 0.19 |
|  | Systolic blood pressure (per 10mm Hg) | -- | -- | -- |  | -0.05 (-0.33, 0.22) | -0.08 (-0.28, 0.11) | 0.84 |
|  | Adiponectin (μg/ml) | -- | -- | -- |  | 0.00 (-0.02, 0.03) | 0.01 (-0.01, 0.03) | 0.53 |
|  | HOMA-IR^a^ | -- | -- | -- |  | -0.03 (-0.39, 0.34) | 0.16 (-0.13, 0.44) | 0.25 |
|  | HDL (mg/dL) | -- | -- | -- |  | 0.01 (-0.01, 0.02) | 0.00 (-0.01, 0.02) | 0.50 |
|  | CRP (mg/L)^a^ | -- | -- | -- |  | -0.10 (-0.26, 0.06) | -0.01 (-0.12, 0.10) | 0.28 |
|  | IL-6 (pg/mL)^a^ | -- | -- | -- |  | -0.08 (-0.35, 0.19) | 0.03 (-0.19, 0.25) | 0.52 |
|  | Leptin (ng/mL)^a^ | -- | -- | -- |  | -0.08 (-0.43, 0.26) | 0.00 (-0.25, 0.25) | 0.47 |
|  | Triglycerides (per 10 mg/dL) | -- | -- | -- |  | -0.01 (-0.10, 0.09) | 0.05 (-0.01, 0.10) | 0.30 |
| ^a^Natural log-transformed | | | | | | | | |
| ^b^P-value for the interaction term between sex and the variable of interest | | | | | | | | |
| ^c^Model includes all variables in model 1 as well as all prenatal characteristics | | | | | | | | |
| ^d^Model includes all variables in models 1 and 2, as well as all early life characteristics | | | | | | | | |
| ^e^Model includes all variables in models 1, 2, and 3. Each early-childhood anthropometric measure was included in a separate model | | | | | | | | |
| ^f^Model includes all variables in models 1, 2, and 3, as well as all mid-childhood characteristics. Mid-childhood BMI-for-age-and-sex z-score was also included in the model. | | | | | | | | |
| ^g^Includes attention deficit/hyperactive disorder (n=6), heart disease (n=4), autism (n=3), chromosomal disorders (n=2), inflammatory bowel disease (n=1), diabetes (n=1), cancer (n=1), and juvenile rheumatoid arthritis (n=1) | | | | | | | | |
| ^h^Model includes all variables in models 1, 2, and 3, and 5. Each anthropometric measure was included in a separate model. | | | | | | | | |
| ^i^Model includes all variables in models 1, 2, 3, and 5, as well as mid-childhood BMI-for-age-and-sex z-score. Each biomarker was included in a separate model. | | | | | | | | |
