# Supplementary material for: Prenatal and childhood predictors of hair cortisol concentration in mid-childhood and early adolescence
Source: PLoS One. 2020 Feb 4;15(2):e0228769. doi: 10.1371/journal.pone.0228769 (PMC6999889; doi:10.1371/journal.pone.0228769)
Supplement: S2 Table — (DOCX) [file pone.0228769.s002.docx]

| **S2 Table. Cross-sectional predictors of mid-childhood hair cortisol concentration^a,b^ in White children** | | | | |
| --- | --- | --- | --- | --- |
|  | Total (n=599) | Males (n=273) | Females (n=326) |  |
| Characteristic | β (95% CI) | β (95% CI) | β (95% CI) | P-interaction^c^ |
| Vigorous physical activity (per 5 hours/week) | -0.03 (-0.22, 0.16) | 0.11 (-0.17, 0.40) | -0.10 (-0.38, 0.17) | 0.24 |
| Youth Healthy Eating Index score (per 10 points) | -0.04 (-0.18, 0.10) | -0.03 (-0.23, 0.16) | -0.03 (-0.23, 0.17) | 0.91 |
| Secondhand smoke exposure (%) | 0.18 (-0.31, 0.66) | 0.22 (-0.35, 0.79) | 0.16 (-0.72, 1.05) | 0.79 |
| Puberty development score | 0.07 (-0.45, 0.59) | -0.01 (-0.91, 0.88) | 0.12 (-0.53, 0.78) | 0.97 |
| Chronic illness^d^ | 0.27 (-0.46, 1.01) | 0.14 (-0.78, 1.06) | 0.57 (-0.69, 1.83) | 0.63 |
| BMI-for-age-and-sex z-score | 0.12 (-0.03, 0.27) | 0.17 (-0.05, 0.39) | 0.07 (-0.15, 0.29) | 0.83 |
| Waist circumference (per 5cm) | 0.09 (-0.02, 0.19) | 0.09 (-0.05, 0.23) | 0.09 (-0.09, 0.26) | 0.73 |
| Height (per 5cm) | 0.01 (-0.12, 0.13) | 0.03 (-0.14, 0.21) | -0.01 (-0.19, 0.17) | 0.94 |
| Waist-height ratio (per 0.1 units) | 0.26 (-0.05, 0.57) | 0.27 (-0.15, 0.69) | 0.25 (-0.23, 0.73) | 0.77 |
| Metabolic risk z-score | 0.13 (-0.19, 0.45) | -0.02 (-0.50, 0.45) | 0.27 (-0.19, 0.72) | 0.53 |
| Systolic blood pressure (per 10mm Hg) | 0.01 (-0.16, 0.18 | -0.03 (-0.27, 0.21) | 0.04 (-0.20, 0.28) | 0.76 |
| Adiponectin (μg/ml) | 0.00 (-0.01, 0.02) | 0.00 (-0.02, 0.03) | 0.00 (-0.02, 0.03) | 0.99 |
| HOMA-IR^a^ | 0.02 (-0.24, 0.28) | -0.13 (-0.49, 0.22) | 0.14 (-0.22, 0.49) | 0.27 |
| HDL (mg/dL) | 0.00 (-0.01, 0.01) | 0.00 (-0.02, 0.02) | 0.00 (-0.02, 0.02) | 0.91 |
| CRP (mg/L)^a^ | -0.01 (-0.12, 0.10) | -0.01 (-0.15, 0.14) | -0.02 (-0.18, 0.14) | 0.96 |
| IL-6 (pg/mL)^a^ | 0.12 (-0.08, 0.31) | 0.08 (-0.18, 0.34) | 0.16 (-0.11, 0.44) | 0.46 |
| Leptin (ng/mL)^a^ | -0.10 (-0.32, 0.12) | -0.22 (-0.56, 0.12) | -0.01 (-0.33, 0.30) | 0.50 |
| Triglycerides (per 10 mg/dL) | 0.02 (-0.03, 0.07) | 0.00 (-0.09, 0.09) | 0.03 (-0.04, 0.10) | 0.82 |
| ^a^Natural log-transformed | | | | |
| ^b^All models adjusted for child age, sex, yearly household income, mother's education (college graduate vs. not a college graduate), maternal age, maternal pre-pregnancy BMI, excessive pregnancy weight gain, maternal smoking during pregnancy, paternal BMI, gestational age, birthweight-for-sex-and-age z-score, breastfed ≥12 months, infant sleep duration, vigorous physical activity, Youth Healthy Eating Index score, secondhand smoking, puberty development score, and chronic illness. Mid-childhood BMI-for-age-and-sex z-score was also included in all models except for waist circumference, height, and waist-height ratio. | | | | |
| ^c^P-value for the interaction term between sex and the variable of interest | | | | |
| ^d^Includes attention deficit/hyperactive disorder (n=6), heart disease (n=4), autism (n=3), chromosomal disorders (n=2), inflammatory bowel disease (n=1), diabetes (n=1), cancer (n=1), and juvenile rheumatoid arthritis (n=1) | | | | |
