# Supplementary material for: Prenatal and childhood predictors of hair cortisol concentration in mid-childhood and early adolescence
Source: PLoS One. 2020 Feb 4;15(2):e0228769. doi: 10.1371/journal.pone.0228769 (PMC6999889; doi:10.1371/journal.pone.0228769)
Supplement: S3 Table — (DOCX) [file pone.0228769.s003.docx]

| **S3 Table. Cross-sectional predictors of early adolescent hair cortisol concentration^a,b^ in White children (n=552)** | | | | |
| --- | --- | --- | --- | --- |
|  | Total | Males (n=241) | Females (n=311) |  |
| Characteristic | β (95% CI) | β (95% CI) | β (95% CI) | P-interaction^c^ |
| BMI-for-age-and-sex z-score | 0.06 (-0.07, 0.19) | 0.04 (-0.17, 0.25) | 0.07 (-0.09, 0.24) | 0.95 |
| Waist circumference (per 5cm) | 0.00 (-0.06, 0.06) | 0.02 (-0.08, 0.11) | -0.02 (-0.10, 0.07) | 0.76 |
| Height (per 5cm) | 0.01 (-0.07, 0.09) | -0.01 (-0.13, 0.11) | 0.07 (-0.04, 0.18) | 0.19 |
| Waist-height ratio (per 0.1 units) | -0.01 (-0.22, 0.21) | 0.08 (-0.25, 0.40) | -0.13 (-0.42, 0.16) | 0.41 |
| ^a^Natural log-transformed | | | | |
| ^b^All models adjusted for child age, sex, yearly household income, mother's education (college graduate vs. not a college graduate), maternal age, maternal pre-pregnancy BMI, excessive pregnancy weight gain, maternal smoking during pregnancy, paternal BMI, gestational age, birthweight-for-sex-and-age z-score, breastfed ≥12 months, infant sleep duration, vigorous physical activity, Youth Healthy Eating Index score, secondhand smoke exposure, and puberty development score, and chronic illness. | | | | |
| ^c^P-value for the interaction term between sex and the variable of interest | | | | |
