# Supplementary material for: Prenatal and childhood predictors of hair cortisol concentration in mid-childhood and early adolescence
Source: PLoS One. 2020 Feb 4;15(2):e0228769. doi: 10.1371/journal.pone.0228769 (PMC6999889; doi:10.1371/journal.pone.0228769)
Supplement: S4 Table — (DOCX) [file pone.0228769.s004.docx]

| **S4 Table. Associations (β [95% CI]) of characteristics with change in hair cortisol concentration^a^ from mid-childhood to early adolescence in White children (n=426) by sex** | | | |  |
| --- | --- | --- | --- | --- |
| **Characteristic** | **Males (n=178)** | **Females (n=248)** | **P-interaction^b^** | |
| **Model 1: Demographic Characteristics** |  |  |  | |
| Child age (per year) | 0.05 (-0.31, 0.41) | 0.36 (0.08, 0.63) | 0.15 | |
| Female | -- | -- |  | |
| Yearly household income (≥ vs. <$70,000) | 0.20 (-0.47, 0.87) | -0.69 (-1.17, -0.22) | 0.03 | |
| Mother's education (college graduate vs. not a college graduate) | -0.25 (-0.97, 0.46) | 0.08 (-0.51, 0.66) | 0.80 | |
| **Model 2: Prenatal Characteristics^c^** |  |  |  | |
| Maternal age (per 5 years) | -0.05 (-0.39, 0.29) | 0.20 (-0.08, 0.47) | 0.30 | |
| Maternal pre-pregnancy BMI (per 5kg/m^2^) | -0.13 (-0.50, 0.24) | 0.17 (-0.06, 0.41) | 0.27 | |
| Excessive pregnancy weight gain | 0.33 (-0.30, 0.96) | -0.11 (-0.53, 0.31) | 0.31 | |
| Mother smoked during pregnancy | -0.88 (-1.78, 0.02) | -0.64 (-1.53, 0.25) | 0.77 | |
| Paternal BMI (per 5kg/m^2^) | 0.05 (-0.32, 0.42) | -0.19 (-0.49, 0.12) | 0.44 | |
| **Model 3: Early Life Characteristics^d^** |  |  |  | |
| Gestational age (per week) | 0.05 (-0.17, 0.28) | 0.00 (-0.13, 0.13) | 0.69 | |
| Birthweight-for-sex-and-gestational age z-score | 0.08 (-0.25, 0.42) | -0.01 (-0.24, 0.23) | 0.46 | |
| Breastfed ≥12 months | -0.18 (-0.84, 0.48) | 0.25 (-0.23, 0.73) | 0.16 | |
| Infant sleep duration (per hour/day) | 0.09 (-0.13, 0.32) | -0.05 (-0.22, 0.12) | 0.14 | |
| **Model 4: Early-Childhood Characteristics^e^** |  |  |  | |
| BMI-for-age-and-sex z-score | 0.01 (-0.30, 0.31) | -0.01 (-0.24, 0.21) | 0.71 | |
| Waist circumference (per 5cm) | -0.10 (-0.54, 0.33) | 0.06 (-0.28, 0.40) | 0.79 | |
| Height (per 5cm) | 0.15 (-0.23, 0.52) | 0.12 (-0.17, 0.42) | 0.75 | |
| Waist-height ratio (per 0.1 units) | -0.45 (-1.35, 0.46) | -0.04 (-0.75, 0.68) | 0.56 | |
| **Model 5: Mid-Childhood Characteristics^f^** |  |  |  | |
| Vigorous physical activity (per 5 hours/week) | -0.02 (-0.53, 0.49) | -0.18 (-0.46, 0.10) | 0.56 | |
| Youth Healthy Eating Index score (per 10 points) | 0.09 (-0.23, 0.40) | -0.12 (-0.34, 0.11) | 0.47 | |
| Secondhand smoke exposure (%) | 0.77 (-0.08, 1.62) | -0.20 (-1.19, 0.80) | 0.17 | |
| Puberty development score | 1.17 (-0.73, 3.06) | -0.19 (-0.88, 0.50) | 0.14 | |
| Chronic illness^g^ | 0.94 (-0.57, 2.44) | 1.49 (0.00, 2.98) | 0.44 | |
| **Model 6: Mid-Childhood Anthropometry^h^** |  |  |  | |
| BMI-for-age-and-sex z-score | -0.07 (-0.42, 0.28) | 0.06 (-0.20, 0.31) | 0.74 | |
| Waist circumference (per 5cm) | -0.09 (-0.33, 0.16) | -0.08 (-0.28, 0.13) | 0.79 | |
| Height (per 5cm) | 0.18 (-0.09, 0.44) | 0.05 (-0.16, 0.27) | 0.96 | |
| Waist-height ratio (per 0.1 units) | -0.48 (-1.20, 0.23) | -0.26 (-0.81, 0.29) | 0.60 | |
| **Model 7: Mid-Childhood Biomarkers^i^** |  |  |  | |
| Metabolic risk z-score | -0.02 (-0.80, 0.75) | -0.01 (-0.51, 0.50) | 0.60 | |
| Systolic blood pressure (per 10mm Hg) | 0.17 (-0.23, 0.57) | -0.06 (-0.34, 0.22) | 0.72 | |
| Adiponectin (μg/ml) | -0.01 (-0.06, 0.03) | 0.00 (-0.03, 0.03) | 0.59 | |
| HOMA-IR^a^ | 0.01 (-0.55, 0.57) | 0.06 (-0.38, 0.48) | 0.66 | |
| HDL (mg/dL) | 0.01 (-0.02, 0.03) | 0.00 (-0.02, 0.02) | 0.76 | |
| CRP (mg/L)^a^ | -0.12 (-0.35, 0.10) | -0.02 (-0.19, 0.15) | 0.41 | |
| IL-6 (pg/mL)^a^ | -0.07 (-0.49, 0.35) | -0.11 (-0.42, 0.19) | 0.78 | |
| Leptin (ng/mL)^a^ | -0.26 (-0.77, 0.25) | 0.08 (-0.26, 0.42) | 0.26 | |
| Triglycerides (per 10 mg/dL) | 0.01 (-0.13, 0.15) | 0.04 (-0.05, 0.12) | 0.63 | |
| ^a^Natural log-transformed | | | |  |
| ^b^P-value for the interaction term between sex and the variable of interest | | | |  |
| ^c^Model includes all variables in model 1 as well as all prenatal characteristics | | | |  |
| ^d^Model includes all variables in models 1 and 2, as well as all early life characteristics | | | |  |
| ^e^Model includes all variables in models 1, 2, and 3. Each early-childhood anthropometric measure was included in a separate model | | | |  |
| ^f^Model includes all variables in models 1, 2, and 3, as well as all mid-childhood characteristics. Mid-childhood BMI-for-age-and-sex z-score was also included in the model. | | | |  |
| ^g^Includes attention deficit/hyperactive disorder (n=3), heart disease (n=3), chromosomal disorders (n=2), autism (n=1), inflammatory bowel disease (n=1), diabetes (n=1), cancer (n=1), and juvenile rheumatoid arthritis (n=1) | | | |  |
| ^h^Model includes all variables in models 1, 2, and 3, and 5. Each anthropometric measure was included in a separate model. | | | |  |
